# Supplementary material for: Diuretic treatment before and after transcatheter aortic valve implantation: A Danish nationwide study
Source: PLoS One. 2023 Mar 16;18(3):e0282636. doi: 10.1371/journal.pone.0282636 (PMC10019742; doi:10.1371/journal.pone.0282636)
Supplement: S1 File — (DOCX) [file pone.0282636.s001.docx]

**SUPPORTING INFORMATION**

**Diuretic treatment before and after Transcatheter Aortic Valve Implantation: A Danish nationwide study**

Xenia Begun, MB,^1^ Jawad H. Butt, MD,^1^ Søren Lund Kristensen, MD, PhD,^1^ Peter E. Weeke, MD, PhD,^1^ Ole De Backer, MD, PhD,^1^ Morten Schou, MD, DMSc,^2^ Lars Køber, MD, DMSc,^1^ Emil L. Fosbøl, MD, PhD^1^

^1^Department of Cardiology, Rigshospitalet, Copenhagen University Hospital, Copenhagen, Denmark.
^2^Department of Cardiology, Herlev-Gentofte University Hospital, Hellerup, Denmark.

**Contents**

# Supplemental Figure 1: Cumulative incidence of death in patients undergoing TAVI and surviving one year with increased, reduced, and unchanged loop diuretic treatment after discharge

Supplemental Figure 2: Changes in loop diuretic groups pre-TAVI and at 1-year post-TAVI in patients with a medical history with heart failure and loop diuretic treatment at TAVI-procedure

# Supplemental Figure 3: Logistic regression of increased loop diuretic treatment in patients undergoing TAVI with a medical history of heart failure and loop diuretic treatment

# **Supplemental Figure 1: Cumulative incidence of death in patients undergoing TAVI and surviving one year with increased, reduced, and unchanged loop diuretic treatment after discharge**

*
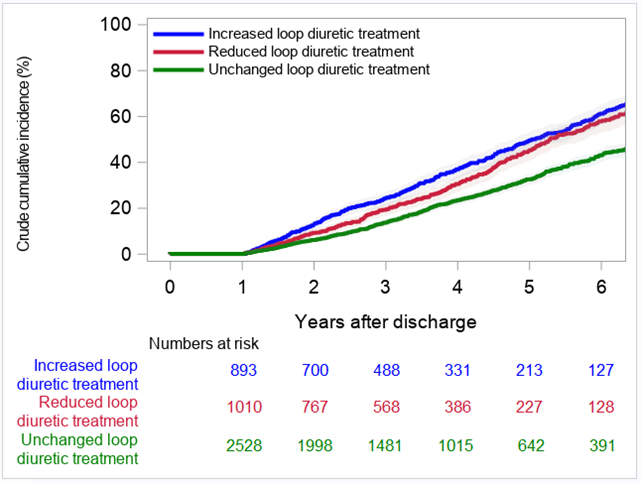
 Abbreviations: TAVI, transcatheter aortic valve implantation.***Supplemental Figure 2: Changes in loop diuretic groups pre-TAVI and at 1-year post-TAVI in patients with a medical history with heart failure and loop diuretic treatment at TAVI-procedure**

*
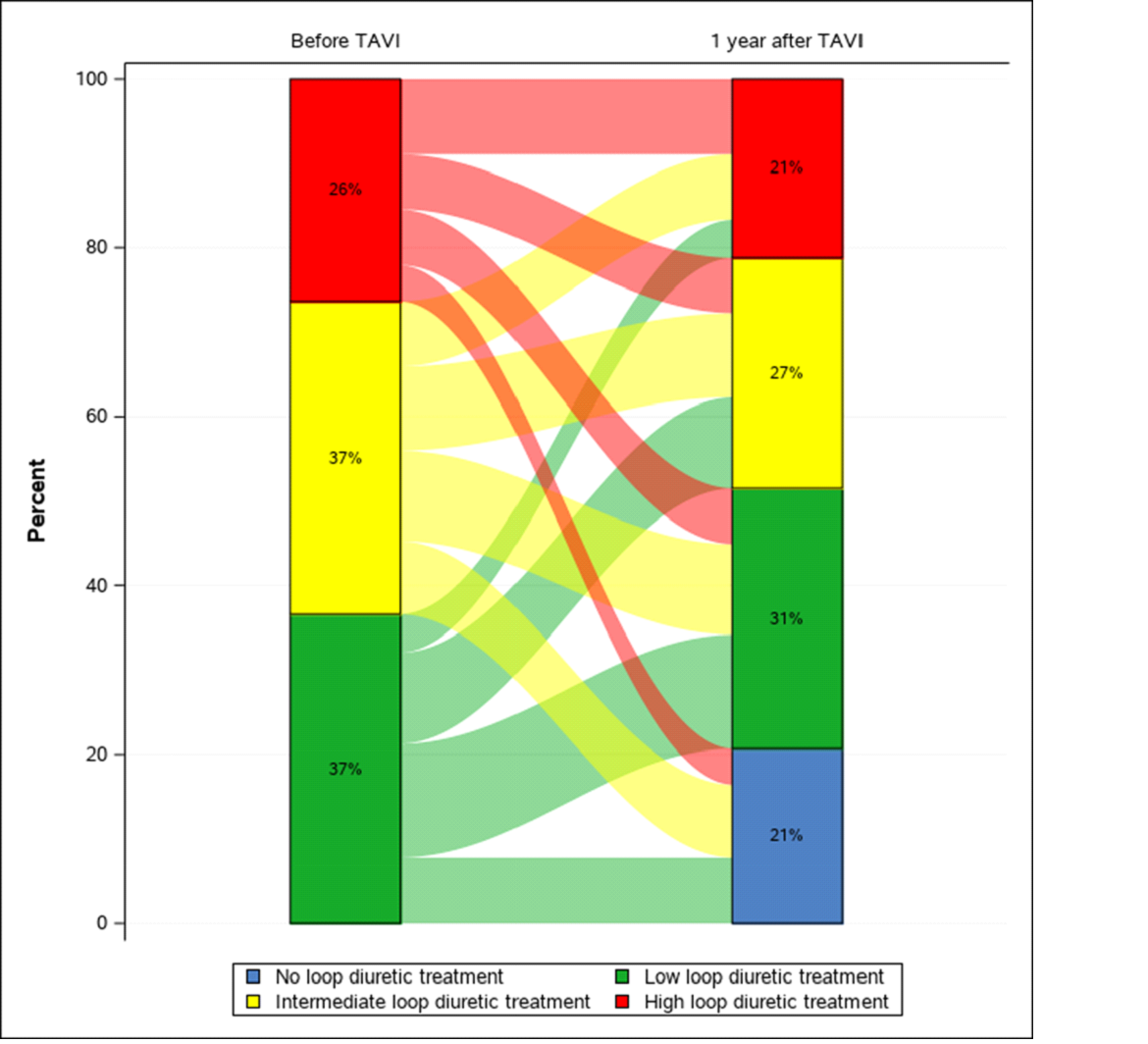
Abbreviations: TAVI, transcatheter aortic valve implantation.
Low loop diuretic treatment 1-40 mg of furosemide (or equivalent bumetanide); intermediate loop diuretic treatment 41-120 mg of furosemide; high loop diuretic treatment >120 mg furosemide.*

# **Supplemental Figure 3: Logistic regression of increased loop diuretic treatment in patients undergoing TAVI with a medical history of heart failure and loop diuretic treatment**

*
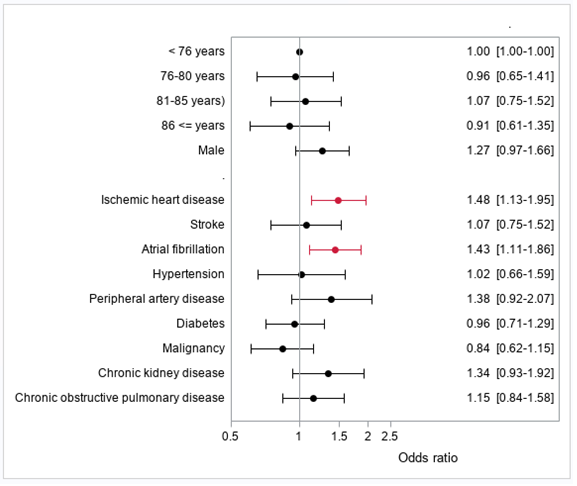
Abbreviations: TAVI, transcatheter aortic valve implantation.*
