# Supplementary material for: Diuretic treatment before and after transcatheter aortic valve implantation: A Danish nationwide study
Source: PLoS One. 2023 Mar 16;18(3):e0282636. doi: 10.1371/journal.pone.0282636 (PMC10019742; doi:10.1371/journal.pone.0282636)
Supplement: Attachment — Submitted filename: Response to Reviewers.docx [file pone.0282636.s002.docx]

**Emily Chenette**

**Editor-in-Chief, PLOS ONE**

January 06, 2023

Manuscript: PONE-D-22-29562, *Diuretic treatment before and after Transcatheter Aortic Valve Implantation: A Danish nationwide study*

Dear Editors and reviewers,

We would like to thank you for the time and effort spent on our manuscript submitted for publication consideration in PLOS ONE. We have responded to all of the reviewers’ comments and adjusted the manuscript accordingly. We believe that the comments have helped improving the manuscript. Please see our point-by-point responses below.

Changes made to the manuscript are designated page and line numbers and are marked in blue text for added text and strike-through for removed text. Two revised versions of the manuscript have also been submitted; one version including track-changes and one version without indication of changes.

Best wishes on behalf of the authors,

Xenia Begun, MB

Department of Cardiology,

Rigshospitalet, Copenhagen University Hospital

Blegdamsvej 9, 2100 Copenhagen, Denmark

E-mail: xeniabegun@gmail.com

**Reviewer #1:**

**In this retrospective, registry based Danish study, the authors studied the value of loop diuretic use as a predictor for 1 year outcome in 4431 pts with severe aortic stenosis surviving 1 year after transcatheter aortic valve implantation (TAVI). Fifty % used diuretics at TAVI and 20% had increased dosage at 1 year. Diuretic use was associated with cardiovascular disease burden. By multivariate survival analysis they found that increased diuretic use at 1 year was significantly associated with poorer outcome. The paper is well written, methods are adequate utilizing well designed health registries in Denmark, thus results seem robust. I have some general comments:**

**Comment no. 1: Study population was all pts surviving 1 year post TAVI, pts who died earlier were excluded (p.6). However, primary outcome was all-cause mortality at 1 year. This is somewhat confusing. Thus I wonder, who were eventually included in the survival analysis? Please explain.**

Our response: Thank you for your comment. It is correct that all patients, who died within the first year post TAVI-discharge were excluded in the study. Hence, index time for the analysis of mortality was set at one year post-TAVI. The primary outcome was all-cause mortality among patients, who survived one year post-procedure and then to one year post this index time, and this has also been specified in the manuscript. We have replaced the figures to the ones shown below. If the editors like the latter version better, we will change this.

Changes made to the manuscript:

**Methods section, p. 7, lines 129-130:**

***Outcomes***

*The primary outcomes were change in loop diuretic treatment pre-TAVI and at 1-year post-TAVI and all-cause mortality* *among patients alive one year after discharge for TAVI procedure.*

***Figure 3: Cumulative incidence of death ~~in patients undergoing TAVI~~ among patients alive one year after discharge for TAVI procedure with increased and reduced/unchanged loop diuretic treatment intensity at 1-year post-TAVI***

*A) Patients ~~undergoing TAVI surviving one year post-procedure~~, alive one year after discharge for TAVI procedure*

*(B) Patients ~~undergoing TAVI surviving one year post-procedure~~, alive one year after discharge for TAVI procedure, excluding patients with no loop diuretic treatment pre-procedure*

**Comment no. 2: Although increased diuretic use was associated with poorer outcome, cardiovascular disease burden is the likely spurious background factor as indicated by Supplemental Table 1 and Fig 3. Was these important background parameters controlled for in the multivariate analysis? If so, what impact did e.g. ischemic heart disease, heart failure etc have on the results?**

Our response: Thank you for your comment. In our multivariable Cox proportional hazard regression model, we adjusted for a history of heart failure and ischemic heart disease. We also adjusted for age (included as a categorical variable: <76 years, 76-80 years, 81-85 years and 86 <= years), year of procedure (included as a categorical variable: 2008-2010, 2011-2013, 2014-2016 and 2017-2019), sex, a history of stroke, atrial fibrillation, hypertension, peripheral vascular disease, diabetes, malignancy, chronic kidney disease, chronic obstructive pulmonary disease, and liver disease. In our adjusted multivariable Cox proportional hazard analysis, we found that increased loop diuretic treatment was associated with a higher risk of death (HR=1.36, 95% CI 1.22-1.52) compared with reduced/unchanged loop diuretic treatment. Additionally, we did a multivariable Cox proportional hazard analysis for patients, who were on diuretic treatment pre-TAVI, and found that increased loop diuretic treatment was associated with a higher risk of death (HR=1.31, 95% CI 1.13-1.52) compared with reduced/unchanged loop diuretic treatment. Hence, adjustment for these factors reduced but not removed the higher risk associated with increased loop diuretic treatment.

In our subgroup analysis, consisting of patients, who had a medical history with HF that were on diuretic treatment at time of TAVI procedure, we also did a multivariable Cox proportional hazard analysis, and found that increased loop diuretic treatment was associated with a higher risk of death (HR=1.33, 95% CI 1.08-1.63) compared with reduced/unchanged loop diuretic treatment. Hence, we tried to adjust our estimates as best as possible and we still found a significant relationship between loop diuretic use and mortality.

**Comment no. 3: In my opinion, Suppl Table 1 and Suppl Fig 3 conveys important information that preferably could be included in the main article.**
Our response: Thank you for your suggestion. We have now included Supplementary Table 1 and Supplementary Figure 3 in the main article, as Table 3 and Figure 5, respectively.

**Comment no. 4: The conclusion in abstract and article should be modified slightly to ensure that the reader is not left with the impression that there is a causal relationship between diuretics use and outcome, at least this is not proven by this study.**

Our response: We appreciate your comment. We have modified the conclusion, so it is not the increased diuretic treatment, which was associated with a higher risk of death, but the associated risk of death was higher in patients with increased diuretic treatment.

Changes made to the manuscript:

**Conclusions:** Among patients undergoing TAVI, surviving one year, one fifth of patients had increased loop diuretic treatment, and a little over one fifth had reduced loop diuretic treatment 1-year post-procedure. ~~Increased loop diuretic treatment was associated with a higher risk of death~~ In patients with increased diuretic treatment, the associated risk of death was higher compared with reduced/unchanged loop diuretic treatment.

**Reviewer #2:**

**Thank you for allowing me to review this very interesting article by Begun and colleagues, titled 'Diuretic treatment before and after Transcatheter Aortic Valve Implantation: A Danish nationwide study'. The authors have used escalation of diuretic therapy as a surrogate marker for symptomatic non-relief, and have gone on to conclude that patients with increased diuretic use in the first year post TAVR, have reduced survival.
I find this article very interesting, and agree with the authors when they say that TAVR may/may not provide symptomatic relief to patients, and it is imperative to analyze this question further, specially with the surgical landscape changing towards TAVR in younger individuals. I have a few questions/comments.**

**Comment no. 1: It would be interesting to know how the echo looked in these patients when they came in for follow-up. The reason i say that is, a significant number of these patients have varying degrees of mitral regurgitation. While the TAVR procedure takes care of the AS, the theory is that the MR should get better over time. But, often that is not the case. And patients with significant MR will have symptoms of dyspnea in the post-op period and may need escalating diureses. So, is the increase in diuresis due to symptomatic non relief from the AS, or progression of the MR, or simply progression of the heart failure? Echo findings would better corroborate your answer.**

Our response: We value this comment, but unfortunately, we do not have access to the echo findings nor to the indication for prescription of diuretic medication. The lack of data on echo findings has now been acknowledged in the Limitations section.

Changes made to the manuscript:

**Limitations section, p. 15, lines 287-289**:

*Furthermore, data on important clinical parameters such as NYHA classification, description of symptom burden, echocardiographic findings and the indication for starting loop diuretic treatment, were not available.*

**Comment no. 2: How many patients left the TAVR room with mild or greater paravalvar regurgitation? How many patients left the TAVR room with a double digit mean gradient across the AV. This may also have a role to play in heart failure admissions post-op.**

Our response: Again, the reviewer raises an important issue, but unfortunately, we do not have access to the clinical data in this retrospective, registry-based study. The lack of data on paravalvular regurgitation and mean gradient have now been acknowledged in the Limitations section.

Changes made to the manuscript:

**Limitations section, p. 15, lines 287-289**:

*Furthermore, data on important clinical parameters such as NYHA classification, description of symptom burden, echocardiographic findings, information on paravalvular regurgitation or mean gradient across the AV, and the indication for starting loop diuretic treatment, were not available.*

**Comment no. 3: 61% of the patients with high diuretic treatment had heart failure. What was the authors definition of heart failure?**

Our response: Thank you for your comment. Comorbidities were obtained by identifying diagnoses during hospitalizations any time before discharge date after the TAVI procedure and admissions were identified by using ICD-8 and ICD-10 diagnosis codes. Heart failure was defined by having a discharge diagnosis consisting of one or more of the codes ICD-in regarding heart failure, cardiomyopathy, hypertensive heart disease or pulmonary edema (diagnosis code DI50, DI42, DJ819, DI110, DI130, DI132, DI425, DI428). This has been validated by another study, and have a very high positive predictive value in the registry (Olesen et al., 2011).

**Comment no. 4: Were their any objective measurements of heart failure? CXR, BNP? Curious because an 80 year old coming in with pneumonia if often also given an extra dose of diuretic since it is assumed that some of the pulmonary symptoms are attributed to diastolic dysfunction.**

Our response: We value this comment, which is important. Unfortunately, we do not have access to objective measurements of heart failure and the indication for diuretic treatment in this study. We agree that this could be the case of increased diuretic treatment, however, to eliminate uncertainties, we have divided loop diuretic treatment into groups according to daily dose of the claimed drug: 1) low dose: 1-40 mg of furosemide or <=1 mg of bumetanide; 2) intermediate: 41-120 mg of furosemide or 2-3 mg bumetanide and 3) high: >120 mg furosemide or >3 mg bumetanide. Additionally, the daily dose was calculated according to the information on the prescriptions from The National Prescription Registry. Five prescriptions prior to the actual prescription were assessed and based on dispensed number of pills and strength of the prescription, a treatment interval was constituted, which was used to estimate the daily dose. However, we have acknowledged the lack of data on objective measurements of heart failure in the Limitations section.

Changes made to the manuscript:

**Limitations section, p. 15, lines 287-289**:

*Furthermore, data on important clinical parameters such as NYHA classification, description of symptom burden,* *echocardiographic findings and objective measurements of heart failure were not available. Neither did we have access to information on paravalvular regurgitation, mean gradient across the AV, nor the indication for starting loop diuretic treatment~~, were not available~~.*

**References**

Olesen, J. B., Lip, G. Y. H., Hansen, M. L., Hansen, P. R., Tolstrup, J. S., Lindhardsen, J., Selmer, C., Ahlehoff, O., Olsen, A. M. S., Gislason, G. H., & Torp-Pedersen, C. (2011). Validation of risk stratification schemes for predicting stroke and thromboembolism in patients with atrial fibrillation: nationwide cohort study. *BMJ (Clinical Research Ed.)*, *342*(7792), 320. https://doi.org/10.1136/BMJ.D124
