# Supplementary material for: Diuretic treatment before and after transcatheter aortic valve implantation: A Danish nationwide study
Source: PLoS One. 2023 Mar 16;18(3):e0282636. doi: 10.1371/journal.pone.0282636 (PMC10019742; doi:10.1371/journal.pone.0282636)
Supplement: Attachment — Submitted filename: Response to Reviewers.docx [file pone.0282636.s003.docx]

**Emily Chenette**

**Editor-in-Chief, PLOS ONE**

February 10, 2023

Manuscript: PONE-D-22-29562, *Diuretic treatment before and after Transcatheter Aortic Valve Implantation: A Danish nationwide study*

Dear Editors and reviewers,

We would like to thank you for the time and effort spent on our manuscript submitted for publication consideration in PLOS One. We have responded to all of the reviewers’ comments and adjusted the manuscript accordingly. We believe that the comments have helped improving the manuscript. Please see our point-by-point responses below.

Changes made to the manuscript are designated page and line numbers and are marked in blue text for added text and strike-through for removed text. Two revised versions of the manuscript have also been submitted; one version including track-changes and one version without indication of changes.

Best wishes on behalf of the authors,

Xenia Begun, MB

Department of Cardiology,

Rigshospitalet, Copenhagen University Hospital

Blegdamsvej 9, 2100 Copenhagen, Denmark

E-mail: xeniabegun@gmail.com

**Reviewer #1:
I thank the authors for their swift and adequate reply to my comments which improved my understanding of this issue. I merely have a couple of suggestions:**

**Comment no. 1: I still think the conclusion in Abstract and main text could be improved somewhat, e.g. instead of "In patients with increased diuretic treatment, the associated risk of death was higher compared with reduced/unchanged loop diuretic treatment" it may be better to simplify to "In patients with increased diuretic treatment, the risk of death was higher compared to those with reduced/unchanged loop diuretic treatment."**
Our response: We would like to thank the reviewer for this comment. We have modified the conclusion to avoid any impression that there is a relationship between diuretics and outcome.

Changes made to the manuscript:

**Conclusions:** Among patients undergoing TAVI, surviving one year, one fifth of patients had increased loop diuretic treatment, and a little over one fifth had reduced loop diuretic treatment 1-year post-procedure. In patients with increased diuretic treatment, ~~the associated risk of death was higher compared with~~ *the risk of death was higher compared to those* with reduced/unchanged loop diuretic treatment.

**Comment no. 2: If pressed for space, Figure 3 B could probably be omitted, and results rather be included in the ms text.**
Our response: We thank the reviewer for this comment. We agree with the reviewer, that this figure is not central in the manuscript. However, we suggest that if editor is pressed for space, we will move the figure to supplemental data.

**Minor: PP12: atrial fibrillation not arterial fibrillation**
Our response: Thank you for this comment. We have corrected the manuscript as shown below.

Changes made to the manuscript:

**Results section, p. 12, lines 215:**
*In adjusted analysis, ischemic heart disease and ~~arterial~~ atrial fibrillation were associated with increased loop diuretic treatment (Supplementary figure 3).*

**Reviewer #2:
The authors need to be congratulated on attempting a nation-wide study to look at diuretic need after TAVR as a marker for worse outcome. The major limitations of this manuscript are that echocardiographic data on post deployment para-valvar leak, or degree of mitral regurgitation was not available, as these can be confounding factors while assessing diuretic need post TAVR.
Nonetheless, I feel this publication has value, and would add to the existing literature.**

Our response: We appreciate this comment made by the reviewer.
